# Immune-related adverse event in the emergency department: methodology of the immune-related emergency disposition index (IrEDi)

**DOI:** 10.1186/s44201-023-00023-y

**Published:** 2024-01-29

**Authors:** Cielito C. Reyes-Gibby, Jeffrey M. Caterino, Christopher J. Coyne, Demetrios N. Kyriacou, Aiham Qdaisat, Jennifer McQuade, Dwight H. Owen, Jason J. Bischof, Sanjay Shete, Sai-Ching Jim Yeung

**Affiliations:** 1 Department of Emergency Medicine, The University of Texas MD Anderson Cancer Center, Houston, TX, USA.; 2 Department of Biostatistics, The University of Texas MD Anderson Cancer Center, Houston, TX, USA.; 3 Departments of Emergency Medicine and Internal Medicine, The Ohio State University Wexner Medical Center, Columbus, OH, USA.; 4 Department of Emergency Medicine, University of California San Diego, San Diego, CA, USA.; 5 Department of Emergency Medicine and Preventive Medicine, Northwestern University Feinberg School of Medicine, Chicago, IL, USA.; 6 Department of Melanoma Medical Oncology, The University of Texas MD Anderson Cancer Center, Houston, TX, USA.

**Keywords:** Immune checkpoint inhibitor, Immune-related adverse events, Oncology, Emergency, Cancer

## Abstract

For many cancer patients, immune checkpoint inhibitors (ICIs) can be life-saving. However, the immune-related adverse events (irAEs) from ICIs can be debilitating and can quickly become severe or even be fatal. Often, irAEs will precipitate visits to the emergency department (ED). Therefore, early recognition and the decision to admit, observe, or discharge these patients from the ED can be key to a cancer patient’s morbidity and mortality. ED clinicians typically make their decision for disposition (admit, observe, or discharge) within 2–6 h from their patient’s ED presentation. However, irAEs are particularly challenging in the ED because of atypical presentations, the absence of classic symptoms, the delayed availability of diagnostic tests during the ED encounter, and the fast pace in the ED setting. At present, there is no single sufficiently large ED data source with clinical, biological, laboratory, and imaging data that will allow for the development of a tool that will guide early recognition and appropriate ED disposition of patients with potential irAEs. We describe an ongoing federally funded project that aims to develop an immune-related emergency disposition index (IrEDi). The project capitalizes on a multi-site collaboration among 4 members of the Comprehensive Oncologic Emergency Research Network (CONCERN): MD Anderson Cancer Center, Ohio State University, Northwestern University, and University of California San Diego. If the aims are achieved, the IrEDi will be the first risk stratification tool derived from a large racial/ethnically and geographically diverse population of cancer patients. The future goal is to validate irEDi in general EDs to improve emergency care of cancer patients on ICIs.

## Introduction

Remarkable advances in the development of monoclonal antibodies that target immune checkpoints are improving clinical response and survival in many kinds of cancer that were previously hard to treat. Immune checkpoint inhibitors (ICIs) bolster a patient’s own immune system to fight the tumor, showing remarkable benefits [1–3]. Immune checkpoints are regulatory pathways that modulate T-cell responses to presented antigens. In healthy individuals, the immune checkpoint proteins mediate self-tolerance and prevent T-cells from attacking normal cells indiscriminately. In cancer patients, multiple gene mutations in their tumor cells produce abnormal proteins that can be recognized as “non-self”, i.e., neo-antigens. Although ICIs confer significant antineoplastic benefits, they also produce a unique spectrum of toxic effects or adverse events, with the disinhibited immune system causing immune-related adverse effects (irAEs) in different organ systems [4, 5]. The use of ICIs is rapidly expanding and evolving with studies seeking optimal ways to combine ICIs with existing treatment modalities and to use ICIs in different clinical settings (adjuvant or neoadjuvant in early-stage disease). Because cancer treatments are typically provided on an outpatient basis, the number of cancer patients who will present to the EDs for treatment and management of irAEs is dramatically increasing. For some patients, multiple organ systems may be affected by ICIs at different times resulting in multiple ED visits, hospitalizations, and even death. We reviewed studies focusing on irAE presentation to the ED. We found 41 case studies and 4 retrospective studies [6–49]. Importantly, one study [22] showed that while there are no differences in the probability of ED presentation by ICI agents (ED visits occurred in 18% vs 21%, pembrolizumab and ipilimumab + nivolumab, respectively, *p* = 0.186), hospitalization rates by ICI significantly varied: patients on single agents had a significantly lower probability of hospitalization (adjusted odds ratio for hospitalization was 0.6 (95% CI = 0.3–0.9; *p* = 0.027) for pembrolizumab versus ipilimumab + nivolumab), suggesting the utility of a tool for risk stratification. Case reports documented ED presentation for irAEs as early as 2 weeks from initiation of ICI. In a cohort study, they found the median time to ED visit was 18 weeks and that as many as 8 irAEs developed in a subset of patients. It is important to note that these studies include small sample size and the need for a prospective study design. IrAEs disrupt treatments and cause morbidity and mortality.

Overall, there is a paucity of studies conducted in the ED that will help guide the care and disposition of these patients. Most ED studies are case studies and are retrospective, with limited samples, without the assessment of variables known to influence the epidemiology of irAEs. Furthermore, the current clinical guidelines for irAEs, while important, have limited utility in the ED since many of the recommendations are based on information that are not available/feasible for use during the ED encounter [50]. They also lack consideration of which specific variables will help in guiding the disposition of patients. To provide appropriate guidance for ED disposition, the full set of data available to the ED physician should be taken into consideration.

We describe an ongoing federally funded project that aims to develop an immune-related emergency disposition index (IrEDi). The project capitalizes on a multi-site collaboration among four members of the Comprehensive Oncologic Emergency Research Network (COCERN): MD Anderson Cancer Center, Ohio State University, Northwestern University, and University of California San Diego. The aims are (1) to develop a probability model (the immune-related emergency disposition index (IrEDi)) to risk stratify patients treated with ICIs for ED disposition and (2) to validate IrEDi using prospective data and determine the predictive validity of IrEDi. If the aims are achieved, the IrEDi will be the first risk stratification tool derived from a large racial/ethnically and geographically diverse population of cancer patients. The future goal is to validate irEDi in general EDs to improve emergency care of cancer patients on ICIs.

## Inflammation and immune biomarkers

C-reactive protein (CRP) is an inflammation biomarker and is readily measured with fast results in most clinical laboratories. Since irAEs of many organ systems produce symptoms that can be non-specific, an increase in CRP can aid the diagnosis of irAE. At the diagnosis of irAE, CRP is increased to above 35 mg/L in over 90% of cases [51]. CRP starts to rise before the onset of clinical symptoms of irAEs. CRP and erythrocyte sedimentation rate (ESR), another readily available inflammation marker may be routinely measured in ED patients with suspected irAE. In a review, Nakamura [52] summarized the biomarkers for irAEs. Among nine factors, the ones that are readily available in the ED are sex, BMI, and absolute lymphocyte count (ALC). Also readily available are the neutrophil-to-lymphocyte ratio (NLR) and platelet-tolymphocyte ratio (PLR), which are markers associated with irAE development [53]. NLR may be correlated with the severity of irAE because the NLR at 2 and 4 weeks after treatment has been shown to predict the response or disease course of irAE [54]. Therefore, the scientific premise of this proposal is that there are promising inflammation/immune biomarkers (C-reactive protein, erythrocyte sedimentation rate, neutrophil-to-lymphocyte ratio/platelet-to-lymphocyte ratio) available during the ED encounter along with epidemiological factors (age, race/ethnicity), biological factors (sex, BMI), cancer (type, stage/metastases) and treatment-related variables (class of ICI, monotherapy versus combination, dose/duration), and clinical status (comorbidities, preexisting autoimmune diseases, vital signs, laboratory results, imaging study results) that may improve the prediction of ED disposition: hospital admission, observation, or discharge. At present, there is no single sufficiently large ED data source with clinical, biological, laboratory, and imaging data that will allow for the development of a clinical tool that will guide early recognition and appropriate ED disposition of patients with potential irAEs. Therefore, patients will be greatly served if IrEDi can be used to facilitate their appropriate/proper ED disposition.

## Methods

### Overarching hypothesis

**Fig. 1:**
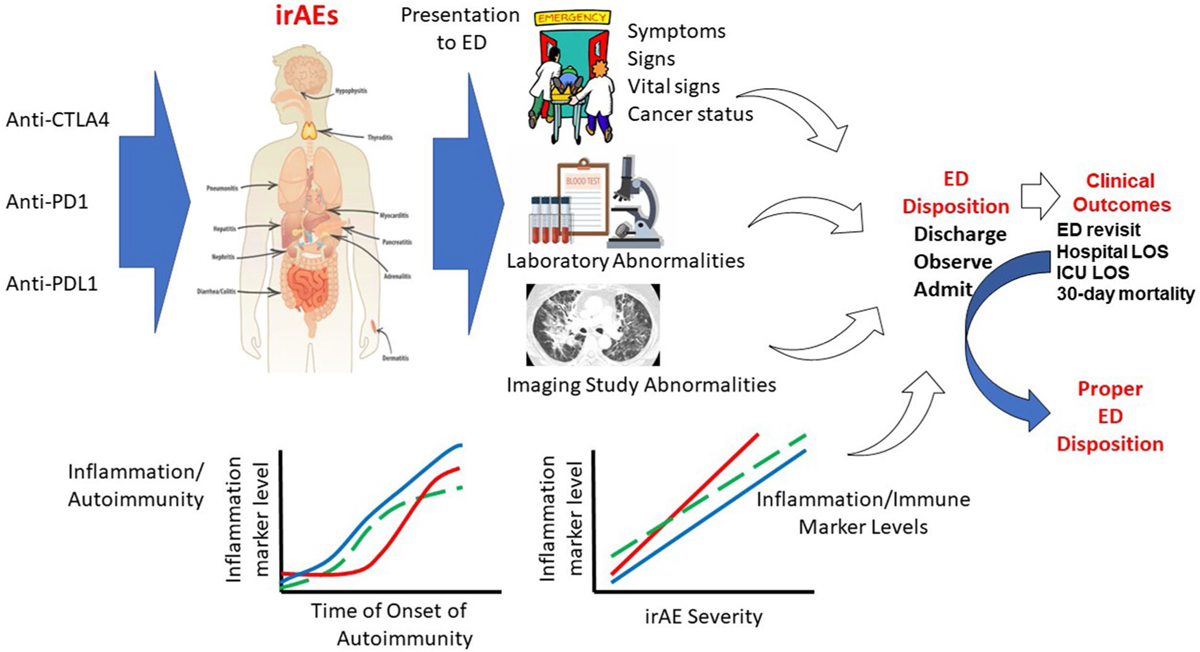
Overarching hypothesis

After cancer patients are treated with ICIs, they may develop irAEs. The symptoms would prompt them to seek medical attention. Depending on the acuity of onset, severity, and availability of clinic visits, these patients may present to the ED. Figure 1 shows our overarching hypothesis that data available during the ED presentation of patients on ICI can determine the proper ED disposition. Immune/inflammation biomarkers rise with the onset of irAE, and the levels correlate with the severity of irAEs. Results of routine and symptom-directed laboratory and diagnostic imaging investigation will inform about the specific irAE and its severity. Vital sign data and cancer status data will inform about the overall clinical status. Data about inflammation/immune biomarkers will inform about the probability and severity of irAE. We hypothesize that while demographic factors, biological variables, cancer and treatment-related variables, vital signs, and laboratory and diagnostic imaging results may grossly predict the clinical course and outcomes, adding inflammation/immune biomarkers available during the ED encounter will improve the prediction of clinical outcome of cancer patients in the ED who have received ICIs. We will derive a probability model (the immune-related emergency disposition index (IrEDi)) to risk stratify patients treated with ICIs for ED disposition using existing data and will validate IrEDi using prospective data. The IrEDi developed in aim 1 will have high sensitivity (≥ 90%) and high specificity (≥ 90%) for predicting appropriate ED disposition (hospital admission, observation, or discharge).

## Research design and methods

### Addressing weaknesses in the rigor of prior research

The following study design for aim 1 (retrospective data) and aim 2 (prospective design) addresses the following weaknesses in the rigor of prior research. To date, studies of irAE in patients presenting to the ED (1) were mainly case studies/case series and used retrospective data, (2) had limited assessment of potential predictor variables and do not include a comprehensive assessment (biological, clinical, laboratory, cancer-related variables), (3) had cross-sectional study design and without follow-up information after ED presentation or after hospital discharge, (4) were mostly conducted in only one institution, (5) had homogenous populations and limited generalizability, (6) had small sample size, and (7) had limited assessment of predictive validity.

We build on the success of the Comprehensive Oncologic Emergency Research Network (CONCERN) and a multi-disciplinary team that has successfully conducted a multi-site cohort study of cancer patients in the emergency setting. Sponsored by the National Cancer Institute and The Office of Emergency Care Research, CONCERN was established in 2015. The PI and sub-award PIs are founding members of CONCERN and have collaborated successfully on a multi-institutional study of cancer ED patients. Dr. Reyes-Gibby, PI (MD Anderson), serves as the Co-Chair of the Scientific Advisory Group of CONCERN; Dr. Yeung is a CONCERN co-PI for MD Anderson; Dr. Caterino, sub-award PI (OSU), is the Founding Chair of CONCERN; Dr. Kyriacou, sub-award PI (Northwestern), and Dr. Coyne, sub-award PI (UCSD), are founding members and serve as site PIs for their ongoing CONCERN projects and all have published together. Future validation of IrEDi will be accomplished in collaboration with a large number of EDs in CONCERN.

### Aim 1: To develop a probability model (the immune-related emergency disposition index (IrEDi)) to risk stratify patients treated with ICIs for ED disposition

We will leverage our existing data (*n* = ~ 2000) of unique ED patients who received ICIs within 3 months of ED presentation at the 4 research sites. We hypothesize that host immune response underlie the development of irAEs and that inflammation/immune biomarkers (C-reactive protein, erythrocyte sedimentation rate, neutrophil-tolymphocyte ratio/platelet-to-lymphocyte ratio) available during the ED encounter will improve the prediction of (a) discharge, (b) observation, and (c) hospital admission, along with traditional factors including epidemiological factors (age, race/ethnicity), biological factors (sex, BMI), cancer (type, stage/metastases) and treatment-related variables (class of ICI, monotherapy versus combination, dose/duration), and clinical status (comorbidities, preexisting autoimmune diseases, vital signs, laboratory results, imaging study results). Table 1 shows our “gold standard” for an appropriate hospital admission. The large sample will also allow for assessing sex as a biological variable and assess racial/ethnic differences.

#### Study design

The study is a retrospective study.

#### Study population

**Fig. 2:**
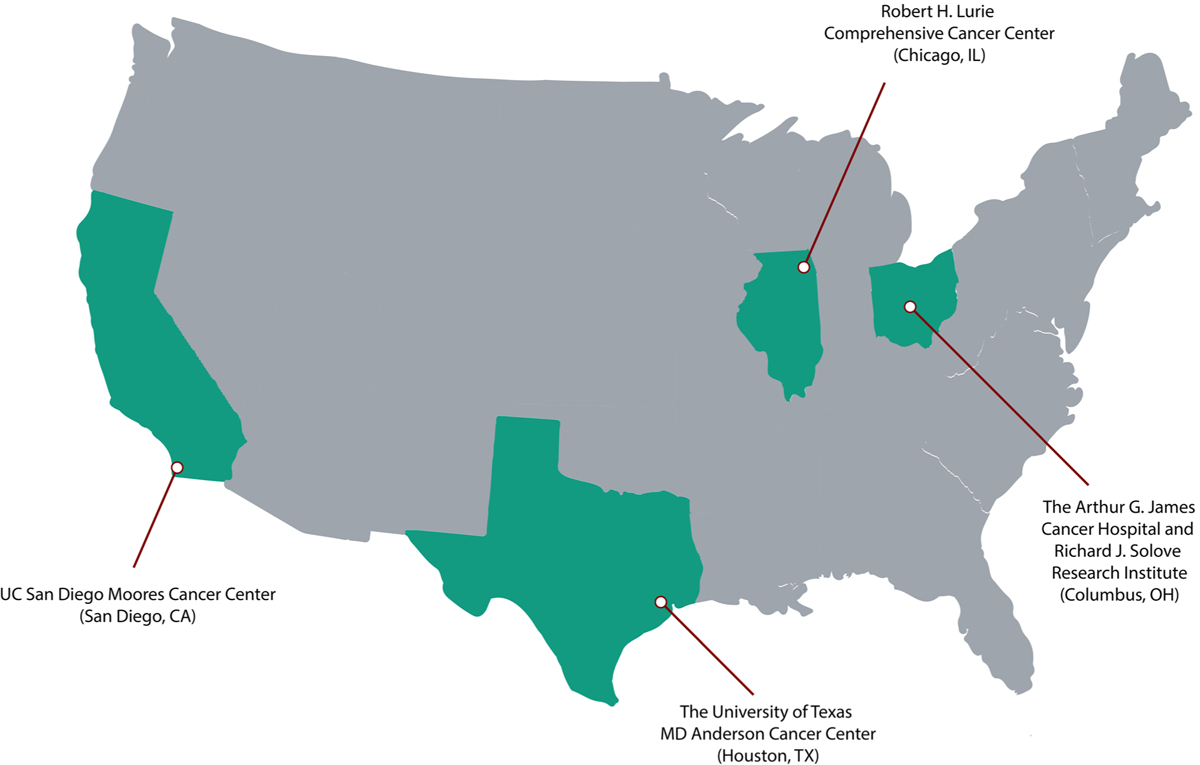
Cancer center study sites

The study population is composed of cancer patients who presented to the ED and had received ICIs within the last 3 months of the ED visit between 1/1/2018 and 11/31/2020 (~ 2000 unique patients with some patients having multiple ED visits). The sites include MD Anderson Cancer Center, Ohio State University, Northwestern University, and University of California San Diego. If the aims are achieved, the IrEDi will be the first risk stratification tool derived from a large racial/ethnically and geographically diverse population (Fig. 2) of cancer patients. All sites use the EPIC electronic medical record (EMR) system.

#### Study variables: our primary outcome variable is ED disposition

EPIC EMR data include whether a patient was admitted to the hospital, observed in the hospital, or discharged to home for a particular ED visit. Other healthcare utilization variables included hospital stay < 48 h, hospital stay > 48 h, and ED revisit within 72 h after ED discharge.

#### We base our gold standard for ED disposition (Table 1) on the “two-midnight rule” and “ED revisit”

Medicare expects patients requiring less than two midnights of hospital care (with few exceptions) to be classified as “observation status” and billed accordingly [55]. Therefore, in this study, a disposition decision of “observation” for a patient who eventually stayed in the hospital for ≥ 48 h will be deemed to be “inappropriate ED disposition,” and a disposition decision of “admission” for patients who eventually stayed in the hospital for < 48 h will also be “inappropriate ED disposition.” A disposition decision of “discharge” for patients who eventually had an ED revisit < 72 h from the date of ED discharge will also be an inappropriate ED disposition. Unscheduled 72-h return ED visit is used as a measure of health care quality based on the commonly held belief that it is very likely to have originated from pre-mature ED discharges. In the general population, 12% of ED revisits within 72 h had adverse events requiring admission [56].

#### Our main predictor variables are inflammation and immune biomarkers

CRP, ESR, PLR, and NLR. Some immune/inflammation-related markers are readily available in the ED. From the CBC results, the neutrophil-to-lymphocyte ratio (NLR) and platelet-to-lymphocyte ratio (PLR) can be calculated: NLR = absolute neutrophil count/the absolute lymphocyte count; PLR = platelet count/the absolute lymphocyte count. ESR and CRP are blood tests that can be rapidly reported and the results will be available to the ED physicians.

Other predictor variables are vital signs, laboratory data, diagnostic imaging studies, comorbidities, sex and obesity, demographic/epidemiologic factors, cancer type, and cancer treatment factors.

#### Statistical analysis approach for aim 1

The primary outcome of the study is ED disposition, which is considered as a categorical variable with three categories: discharge, observation, and hospital admission. Descriptive analyses will be conducted for the primary outcome, as well as our main predictor variables of interest (e.g., inflammation biomarkers) along with treatment variables, cancer type, and demographics. Descriptive statistics, such as mean, standard deviation (SD), median, quartiles, range, frequencies and proportions, and confidence intervals (CI), as well as graphical presentations (e.g., boxplot), will be assessed. Univariate associations between the outcome and various factors will be evaluated using ANOVA/*t*-test or chi-squared test. We will use non-parametric tests (e.g., Kruskal–Wallis, Mann–Whitney, Fisher’s exact test) when appropriate. All analyses will be performed at a two-sided significance level of 0.05 unless otherwise specified. We will use a false discovery rate-based approach to account for multiple comparisons where appropriate.

#### The goal is to develop a probability model (the immune-related emergency disposition index (IrEDi)) to risk stratify patients treated with ICIs for ED disposition

**Fig. 3:**
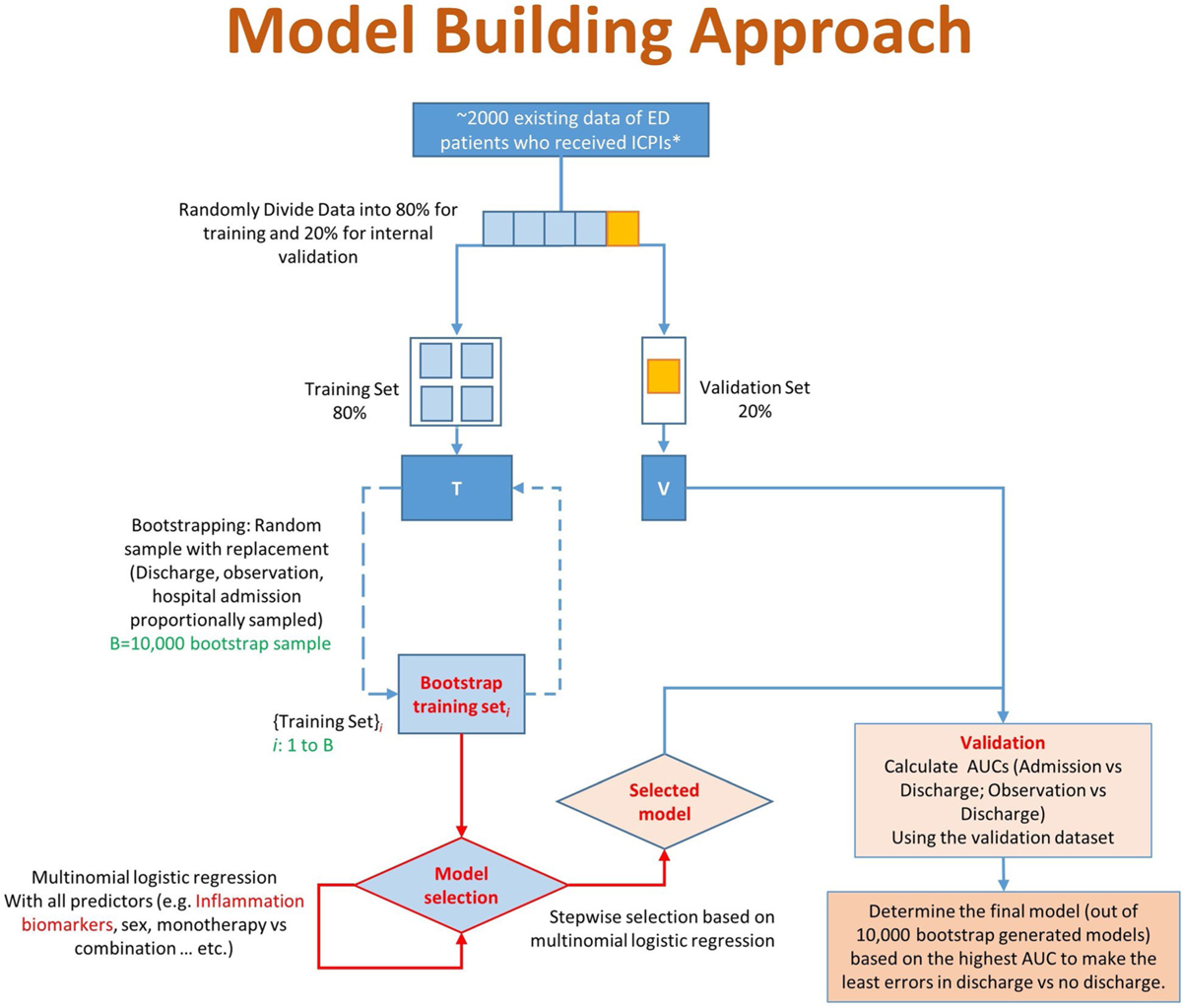
Model-building approach

Therefore, the gold standard ED disposition (hospital admission, observation, or discharge) discussed in Table 1 is our primary outcome. The potential predictors of interest include inflammation/immune biomarkers (e.g., ESR, CRP, NLR, PLR), biological (e.g., sex, age), epidemiological (e.g., race/ethnicity, BMI), and clinical factors (e.g., monotherapy vs combination). To develop the probability model, we will model the multilevel responses using multinomial logistic regression. In the multinomial model, we will consider “discharge” as the reference category and compare “observation” and “hospital admission” with the reference category. We will use a generalized logit link function to evaluate the significance of the predictors as such a link function is of the most general form and does not have specific order assumptions. Odds ratios and 95% CIs will be reported. Collinearity between predictors will be examined, and highly collinear variables will be removed from the model while keeping the variables with the highest *r*-square value. Our model-building approach is depicted in Fig. 3.

We will first divide the data into five parts: training (80% of the data, 4 parts) and validation (20% of the data, 1 part). The training data will be further resampled 10,000 times with replacement (Bootstrap) while keeping the proportions of discharge, observation, and hospital admission intact within each of the bootstrap samples. For each bootstrap sample, we will perform multinomial logistic regression that will include all the predictors and select a model using a stepwise variable selection process. We will then calculate the operating characteristics (e.g., AUC) of the selected model using the separately kept validation dataset. The final model out of 10,000 so-generated boostrap models will be selected based on the clinical utility of the model (i.e., not to discharge patients with potentially serious irAEs, the model will be selected based on the highest AUCs to make the least errors in predicting discharge versus not discharged). The entire process will be repeated by using different four parts (out of the original 5 parts) as training and the remaining part as a validation dataset. The final IrEDi will be averaged over all such models. If the IrEDI generated is not valuable in making correct decisions for dispositions (based on sensitivity and specificity), then we will evaluate alternate strategies for model building such as machine learning or random forest.

The sample size justification was conducted based on the univariate multinomial logistic regression analysis. We considered both continuous and binary predictors. In particular, for the purpose of sample size justification, we considered platelet-to-lymphocyte ratio (PLR) as an example of continuous predictors and sex as an example of binary predictor. Based on our existing data of ED patients, there are ~ 1200, ~ 600, and ~ 200 patients respectively for different ED disposition outcomes, hospital admission, and discharge and observation. For PLR, the pooled SD was obtained as 270 from our preliminary data. Assuming the means of PLR were 300, 255, and 285 respectively for patients in hospital admission, discharge, and observation categories, we will have ~ 85% power to detect the difference in the means with a significance level of 0.05 using one-way ANOVA. For sex, we assumed that the proportions of different ED disposition outcomes in males were 8%, 29%, and 63% respectively corresponding to observation, discharge, and hospital admission categories and assumed that the proportions in females were 12%, 31%, and 57%, respectively. When comparing the multinomial proportions of different outcomes between males and females, we will have ~ 85% power to detect the difference in distributions of ED disposition outcomes between the two groups with a significance level of 0.05 using the chi-square test for proportions in three levels. Sample size justification was conducted using East 6 [57] (statistical software by Cytel Inc., Cambridge, MA). Thus, the study sample is adequate to generate IrEDi.

#### Expected outcome

Our hypothesis is that the inflammation/immune biomarkers (CRP, ESR, NLR, and PLR) will improve the prediction of appropriate ED disposition along with traditional predictive factors. Through the boot-strapping model building, we will examine whether any or ≥ 1 inflammation/immune biomarker will be a significant predictor in the best prediction model. Having ≥ 1 inflammation/immune biomarker as a significant predictor in the best prediction model will confirm our hypothesis. However, irrespective of whether our hypothesis is proven or rejected, this work will deliver a probability model (IrEDi) that may be clinically useful in oncologic emergency clinical care to guide the ED disposition of cancer patients treated with ICIs, including the importance of sex and race/ethnicity.

### Aim 2: To validate IrEDi using prospective data and determine the predictive validity of IrEDi

#### Study design

This is a prospective observational study. We will conduct a prospective cohort study of cancer patients presenting to the ED, who have received ICIs within 3 months prior to the index ED visit. This multi-center study will involve the 4 participating CONCERN sites stated above, recruiting a total of 1200 patients over a 3-year period. The ED physician for the study patients will make the ED disposition decision without knowing the IrEDi score. A common limitation of ED studies is the likelihood that patients may not present to the same EDs for all medical emergencies or acute care. Thus, we will conduct follow-up telephone calls 30 days after the index ED visit to assess ED revisits and to obtain outcome data (e.g., hospitalization) within 30 days of the index ED visit. The appropriate ED disposition will be determined in the same manner as described in aim 1. We expect that the IrEDi probability model to be validated by this multi-center prospective cohort to have high (> 0.90) sensitivity and specificity in the prediction of appropriate ED disposition. We will use electronic EMRs from the 4 CONCERN participating sites (MD Anderson, Ohio State University, Northwestern University, and University of California in San Diego). Each site uses EPIC. Recruitment and data collection will be standardized across sites with MD Anderson serving as the central site for data analyses. Patient recruitment will start on year 1 and will be over a 3-year period with a total recruitment of 1500 patients for all sites. Each site will recruit at least 300 patients. All ED sites are NCI-designated Comprehensive Cancer Centers.

#### Study population

Patient eligibility and exclusion criteria are shown in Table 2. Our latest available information for each site describes the potential study sample of adult cancer patients who received ICIs in the year 2019. In our experience, cancer patients have a high rate of participation and a low rate of loss to follow-up. Even with a conservative estimate that 20% may refuse to participate and 10% will be lost to follow-up, with the expected dramatic increase in the use of ICIs in cancer patients, we are confident that we will accrue 1500 evaluable patients for this study with 30-day follow-up information. We have successfully conducted a prospective observational cohort study in CONCERN and published the results [58–60], thus demonstrating a track record of successful collaboration. Based on the demographic characteristics of the current patient population at each site, we expect to have a diverse population. For example, OSU sees as many as 35% African-Americans whereas UCSD sees as many as 20% Hispanic patients. We have also powered our study to recruit 40% females therefore ensuring analyses of sex as a biological variable (please see the statistical analyses section).

#### Patient recruitment and retention

Since the study involves minimal risk (not involving drug therapy or intervention), the PI delegated research staff will introduce the study and if a potentially eligible subject is unable to be consented in person, they may be verbally consented over the phone. In this instance, the informed consent will be a verbal consent. A member of the study team will identify potentially eligible subjects from the daily ED census.

#### Data collection

Research staff interaction with the patients in the ED will occur at two time points: during the patient’s ED visit and at the 30-day post-ED visit telephone follow-up. After obtaining informed consent (each enrolled patient will be registered in the respective institution’s protocol enrollment system). The 30-day follow-up interview will be conducted by the respective research staff at each institution. Patient interview will occur in 30 ± 3 days after ED disposition to determine ED-related outcomes (ED revisit and hospitalization).

#### EPIC data

All other information will be from EPIC EMR as described in aim 1.

#### Statistical analysis for aim 2

Aim 2 is to validate the probability model (IrEDi) of ED disposition developed in aim 1 using data from a prospective cohort (*n* = 1500). We will use Table 1, the appropriate ED disposition as the gold standard outcome to assess the predictive performance of IrEDi. We will calculate the specificity and sensitivity of the IrEDi by constructing the receiver-operating characteristic curve (ROC) and use the area under the curve (AUC) to estimate the predictive ability of IrEDi to discriminate between patients from different categories (e.g., discharge, observation, hospital admission). We will also consider measures such as sensitivity, specificity, positive predictive value, accuracy, C-index, *D* statistics, and negative predictive value to assess the correct decision for disposition using the IrEDi. Additionally, partial area under the curve will be calculated for areas with high specificity (e.g., ≥ 95%). The purpose of aim 2 is to provide valid predictive value of IrEDi in a new prospective cohort of ED patients who have received ICIs. Although IrEDi developed in aim 1 will have undergone internal validation, the external validation (aim 2) for new patients seen at different sites in the USA would allow generalizability and clinical implementation of the IrEDi.

With a projected sample size of 1500 new patients in aim 2, based on preliminary data of ED patients, we expect there will be ~ 900, ~ 450, and ~ 150 ED patients respectively for hospital admission, discharge, and observation. Using the prospective data, the margin of error for the 95% confidence interval for the AUC will be within 0.06 units.

If missing data and/or drop-outs become an issue (i.e., > 5% of patients), we will examine whether participants who are lost to follow-up differ from those who continue to be in the study. In the analysis, we will adjust for the covariates that are found to be related to missingness and potentially related to the outcomes, which might mitigate the impact due to potential missing-notat-random (MNAR) mechanisms [61]. We will also conduct additional sensitivity analyses using a variety of approaches, i.e., multiple imputation, pattern-mixture models, and selection models, to account for potential missing-at-random or MNAR mechanisms [61, 62].

#### Expected outcome

This is an independent validation of IrEDi using a multi-center prospective observational cohort. We expect that this cohort will perform similarly to the internal validation cohort in aim 1. Therefore, we expect to validate IrEDi for use in general EDs for the care of cancer patients treated with ICIs and to develop an EPIC-based software that will auto-generate a display of IrEDi predictive scores for use in a fast-paced ED setting.

#### Challenges and potential pitfalls

Challenge #1: We acknowledge that among the limitations of this study is that a number of contributing factors may affect the ultimate ED disposition (including access to follow-up health care; ability to fill medication prescriptions; level of functional independence or ability to ambulate; ability of the patient to care for himself or herself at home; family and social support network; insurance, availability of beds; staffing). The assessment of these factors is outside the scope of this proposal. This proposal will develop a predictive tool that focuses on the patient’s clinical status and clinical data available during the ED presentation. Predictive tools can guide ED physicians in making the correct decision for disposition. Challenge #2: Definitive diagnosis of irAE is often not possible in the ED. Making decisions on care, diagnosis, and treatment with limited information and time is a major challenge in oncologic emergencies. Without a definitive diagnosis of irAE, we only rely on information and data available in the ED to develop a probability prediction model to predict the appropriate ED disposition. Challenge #3: Patients present to the ED for complications, etc. of their disease or treatment, and by nature, we will have a heterogeneous population of cancer patients with different types of cancer. Therefore, we will incorporate these variables as covariates. Challenge #4: Patients may be missed if the recruitment staff is not scheduled 24/7 as the ED. Since the ED EPIC schedule is reviewed each day and we may take verbal consent, the research staff may call the patient to elicit participation in the study and their EPIC data will be accessed and a 30-day follow-up call may be initiated.

Pitfall #1: Missing data and loss to telephone follow-up could be a challenge. If a patient fails to respond to our follow-up via telephone after 3 attempts, the patient will be considered as a loss to follow-up. We have powered our study to accommodate loss to follow-up. We will also apply data imputation methods, if relevant assumptions hold. Pitfall #2: As above, if missing data and/or drop-outs become an issue, we will adjust for the covariates that are found to be related to missingness and potentially related to the outcomes, which might mitigate the impact due to potential MNAR mechanisms [61] and also conduct sensitivity analyses, i.e., multiple imputation, pattern-mixture models and selection models [61, 62]. Pitfall #3: If IrEDi generated in aim 1 has poor predictive performance and is not valuable in making correct decisions for dispositions (based on sensitivity and specificity), then we will evaluate alternate strategies for model building such as machine learning or random forest. Pitfall #4: If the original irEDi derived from aim 1 fails to be validated by the new prospective data from aim 2, a new revised IrEDi scoring system can be developed using the pooled data from both aims and using a training dataset consisting of randomly selected 80% and a validation dataset consisting of the remaining 20%.

## Conclusions

While ICIs have given hope to many patients, they unfortunately can also cause irAEs. irAEs are particularly challenging in the ED because of atypical presentations, the absence of classic symptoms, the delayed availability of diagnostic tests during the ED encounter, and the fast pace in the ED setting. At present, there is no single sufficiently large ED data source with clinical, biological, laboratory, and imaging data that will allow for the development of a clinical tool that will guide the appropriate ED disposition of patients with potential irAEs from ICIs. To our knowledge, this project will be the largest cohort study of a geographically, racially, and ethnically diverse population of adult ED patients receiving ICIs. With ICIs being used in the advanced stage of many cancers and its expansion as adjuvant and neoadjuvant in the early stage of disease, we will have the population required for this study to comprehensively assess relevant variables and will also ensure the assessment of sex as a biological variable and assess racial/ethnic differences. This study is also the first to conduct follow-up calls of ED patients in 30 days, addressing a common limitation of our understanding of the outcomes of ED patients (i.e., visiting a different ED or being admitted to a different hospital after the ED visit). The prospective study design will also allow for the assessment of the predictive validity of IrEDi. Thus, patients would be greatly served if IrEDi can be used to facilitate their appropriate ED disposition. Future projects are (1) to validate the IrEDI for use in general EDs and (2) to develop an electronic medical record system-based software that will auto-generate a display of IrEDi predictive scores for use in a fast-paced ED setting.

## Figures and Tables

**Table 1:** The gold standard ED disposition

ED disposition	Healthcare utilization
Discharge	No ED revisit within 72 h of ED discharge
**Discharge**	ED revisit within 72 h of ED discharge
**Observation**	Hospitalized for ≥ 48 h
Observation	Hospitalized for < 48 h
Admission	Hospitalized for ≥ 48 h
**Admission**	Hospitalized for < 48 h

Inappropriate ED dispositions are in bold

**Table 2:** Eligibility and exclusion criteria

Eligibility criteria:
Patient seeking care in the emergency department Cancer diagnosis (excluding non-melanoma skin cancer) Age ≥ 18 years History of receiving ICI either as monotherapy or in combination within the last 3 months Speaks English or Spanish Patient agrees for follow-up phone call 30 days after ED disposition Able to understand the description of the study and give written informed consent
Exclusion criteria: Pregnant Unable to give consent Refusal of follow-up phone call
